# Psychosocial Factors of Antenatal Anxiety and Depression in Pakistan: Is Social Support a Mediator?

**DOI:** 10.1371/journal.pone.0116510

**Published:** 2015-01-28

**Authors:** Ahmed Waqas, Nahal Raza, Haneen Wajid Lodhi, Zerwah Muhammad, Mehak Jamal, Abdul Rehman

**Affiliations:** 1 CMH-Lahore Medical College and Institute of Dentistry, Shami Road, Lahore Cantt, Pakistan; 2 Allama Iqbal Medical College, Lahore, Pakistan; Medical University of Vienna, AUSTRIA

## Abstract

**Introduction:**

Pregnancy is generally viewed as a time of fulfillment and joy; however, for many women it can be a stressful event. In South Asia it is associated with cultural stigmas revolving around gender discrimination, abnormal births and genetic abnormalities.

**Methodology:**

This cross-sectional study was done at four teaching hospitals in Lahore from February, 2014 to June, 2014. A total of 500 pregnant women seen at hospital obstetrics and gynecology departments were interviewed with a questionnaire consisting of three sections: demographics, the Hospital Anxiety and Depression Scale (HADS) and the Social Provisions Scale (SPS). Pearson’s chi-squared test, bivariate correlations and multiple linear regression were used to analyze associations between the independent variables and scores on the HADS and SPS.

**Results:**

Mean age among the 500 respondents was 27.41 years (5.65). Anxiety levels in participants were categorized as normal (145 women, 29%), borderline (110, 22%) or anxious (245, 49%). Depression levels were categorized as normal (218 women, 43.6%), borderline (123, 24.6%) or depressed (159, 31.8%). Inferential analysis revealed that higher HADS scores were significantly associated with lower scores on the SPS, rural background, history of harassment, abortion, cesarean delivery and unplanned pregnancies (P < .05). Social support (SPS score) mediated the relationship between the total number of children, gender of previous children and HADS score. Women with more daughters were significantly more likely to score higher on the HADS and lower on the SPS, whereas higher numbers of sons were associated with the opposite trends in the scores (P < .05).

**Conclusion:**

Because of the predominantly patriarchal sociocultural context in Pakistan, the predictors of antenatal anxiety and depression may differ from those in developed countries. We therefore suggest that interventions designed and implemented to reduce antenatal anxiety and depression should take into account these unique factors.

## Introduction

In recent years much has been published on the psychological science of pregnancy. Although pregnancy is generally viewed as a time of fulfillment and joy, for many women it can be a stressful event. In our part of the world, South Asia, it is associated with cultural stigmas revolving around gender discrimination, abnormal births and genetic abnormalities. It is also associated with several psychiatric problems in women, most notably depression and anxiety.

Around the globe, studies have shown a high prevalence of psychiatric illness in pregnant women. Estimates of the prevalence of antenatal depression and anxiety vary. Gaynes et al., in a systematic review of 109 articles published in English between 1980 and 2004, found that up to 13% of pregnant women suffer from major or minor depression [1]. Faisal-Cury et al. reported a higher prevalence of depression (20% according to the Beck Depression Inventory) and anxiety (60% according to the State-Trait Anxiety Inventory) among pregnant women in Sao Paulo, Brazil in 2007 [2]. Owing to gender sensitivities in the cultural setting of South Asia, an especially high prevalence of psychiatric illnesses in pregnant females has been reported. For example, a study in rural Bangladesh in 2011 estimated an 18% prevalence of antenatal depression and a 29% prevalence of antenatal anxiety, [3] and a 2006 study in Karachi, Pakistan reported a 34% prevalence of antenatal depression [4]. In their comparison of antenatal depression among Pakistani and Canadian women, Mahboob et al. found a higher prevalence of antenatal depression among Pakistani women (48%) than Canadian aboriginal (31%) and Caucasian (9%) women [5]. These South Asian studies used the Edinburgh Postnatal Depression Scale (EPDS) to screen for antenatal depression.

Several studies have drawn attention to the adverse effects of antenatal anxiety and depression in the developing child. These effects include preterm birth [6,7], low birth weight [6,8], reduced cognitive ability and increased fearfulness [9], increased incidence of respiratory and skin illnesses in early life [10] and elevated awakening cortisol levels [11]. Moreover, in their literature review Kinsella et al. concluded that fetal heart rate, activity, sleep patterns and movements, all indicators of neurobehavioral development, were significantly affected by maternal stress, depression and anxiety [12]. Antenatal depression is also the strongest predictor of postnatal depression [13], which is itself associated with several adverse effects in the infant.

In the past decade, research has actively focused on elucidating the underlying causes of antenatal anxiety and depression. Antenatal depression has been found to be associated with domestic violence [14,15], low social support [13,16,17], social conflict [16], low income [17], antenatal anxiety [17,18], unwanted pregnancy [18,19], history of depression [13,18,19] and previous prenatal loss [20,21], while antenatal anxiety has been associated with less positive attitudes towards pregnancy, low income, low educational level, low marital satisfaction, low social support, longer duration of infertility and history of treatment failure with assisted reproductive technologies [22–24]. Similar risk factors have been reported in various studies in Pakistan [25–27].

Because of the cultural and socioeconomic environment in various developing regions of the world, several unique factors contribute to antenatal anxiety and depression in these regions. South Asia is among the most densely populated and poorest regions in the world, and it faces huge social, economic and health challenges. Most South Asian societies are patriarchal and characterized by discrimination against women. It is generally considered more desirable to have male offspring than female offspring [28,29]. Owing to cultural stigmas and gender discrimination, males enjoy better access to health facilities, education and employment. Qadir et al. have pointed out that this gender disadvantage is strongly associated with psychological morbidity among women in Pakistan [30]. Indeed, the prevalence of depression and stress in Pakistan has been found to be far greater in women than in men [31]. Whether gender discrimination and the preference for sons rather than daughters contribute to depression and anxiety among pregnant women is not known, and to our knowledge, no study has been conducted to clarify this relationship. Thus the purpose of our study was to bridge this gap in scientific knowledge by investigating the factors associated with antenatal depression and anxiety, with particular emphasis on the association between gender discrimination, the preference for sons, and mental health in pregnant woman.

## Methodology

This cross-sectional study was carried out at four teaching hospitals in Lahore from February, 2014 to June, 2014: the teaching hospital of CMH Lahore Medical College and Institute of Dentistry, Jinnah Hospital, Services Hospital and Lady Willingdon Hospital. The study was approved by the Ethics Review Committee of CMH Lahore Medical College and Institute of Dentistry, Lahore (CMH LMC).

Pregnant women who came to the obstetrics and gynecology departments spontaneously for routine prenatal or perinatal care were included in this study. We included only those women whose socioeconomic level was characterized as low or lower-middle income.

The data were collected by convenience sampling since we could not ensure random sampling due to lack of resources. Each woman was interviewed by one of four fourth-year medical student enrolled at CMH LMC. All four students took part in a 2-day interviewing skills workshop at the Department of Psychology, CMH LMC. The workshop was run by experienced psychologists employed at this department. Training was considered necessary due to the sensitive nature of the questions asked during the questionnaire-guided interview.

The women were informed about the objectives of the survey and ensured anonymity. Only women who were willing to participate in the survey were interviewed. Written informed consent was provided by each of the 500 participants who agreed to take part.

The questionnaire consisted of three sections: demographics, the Hospital Anxiety and Depression Scale (HADS) [32] and the Social Provisions Scale (SPS) [33]. In the demographics section, participants were asked about their age, ethnicity, education, background, occupation, any history of miscarriage, abortion, harassment, number of cesarean deliveries and whether their present pregnancy was planned or unplanned. The total number of children, their gender and ages were also recorded.

The second part of the questionnaire consisted of the Urdu translation of the HADS. According to a systematic review published in 2007, the HADS has been rigorously evaluated for cross-cultural and criterion validity in Pakistan [34]. This psychological instrument is widely used to screen for anxiety and depression. It consists of two subscales designed for anxiety and depression separately. Each subscale yields a score ranging from 0 to 21, with increasing scores associated with higher levels of anxiety and depression. These scores are divided into three categories: 0–7 = normal, 8–10 = borderline abnormal (borderline case) and 11–21 = abnormal (case).

The third part of the questionnaire consisted of the Urdu translation of the Social Provision Scale [33]. This instrument assesses perceived social support and consists of 24 questions with a Likert-type, 4-point response scale ranging from 1 (strongly disagree) to 4 (strongly agree). Each statement describes an aspect of the participant’s current social network. This scale assesses six types of social relationships including guidance (advice or information), reliable alliances (assurance that others can be counted on in times of stress), reassurance of worth (recognition of one’s competence), attachment (emotional closeness), social integration (a sense of belonging to a group of friends), and opportunities for nurturance (providing assistance to others) [33]. For the purpose of analysis, the total SPS score can also be used.

All data were analyzed with the SPSS (v. 20.) Frequencies and descriptive statistics were analyzed for demographic variables and categories of the HADS subscales. The data were plotted on a histogram to assess normality. Bivariate correlations were used to identify associations between demographic characteristics, scores on the HADS subscales and scores on the SPS. Linear regression was used to analyze associations between the numbers of sons and daughters (as dichotomous variables) and depression subscale scores. The dichotomous variable for number of sons was coded as pregnant women with no sons (0) or with 1 or more sons (1). Similarly, the dichotomous variable for number of daughters was coded as pregnant women with 0 or 1 daughter (0) or more than 1 daughter (1). These dichotomous variables were entered in an initial regression model (Model 1), then SPS scores were entered to analyze their effect on the variables in the first model (Model 2).

## Results

A total of 500 women participated in the survey. Their mean age was 27.41 years (5.65), and their ethnic distribution was Punjabi 369 (73.8%), Urdu-speaking 110 (22%) and other 21 (4.2%). Self-reported educational level was 85 (17%) illiterate, 315 (63%) high school, 60 (12%) intermediate and 40 (8%) university-level. Most of the respondents were housewives (441 women, 88.2%) and 59 (11.8%) were employed outside the home. Most of the respondents had an urban background (208, 41.6%) followed by a rural (182, 36.4%) and semiurban background (110, 22%). Most respondents were from the lower-middle (284, 56.8%), lower (148, 29.6%) or middle class (68, 13.6%). Their current pregnancy was planned according to 135 respondents (27%) and unplanned according to 365 (73%). Previous miscarriage was reported by 44 women (8.8%), and previous abortion by 110 (22%). Harassment had been experienced by 33 (6.6%) of the respondents. The mean number of children in our sample of respondents was 1.5 (1.42). A history of at least one episiotomy was reported by 81 women (16.2%), and a history of at least one cesarean delivery was reported by 136 (27.2%).

On the HADS, the mean anxiety score was 9.71 (4.24) and the mean depression scores was 7.85 (4.03). Mean score on the SPS was 72.3 (12.2). Anxiety levels in the participants were categorized as normal in 145 (29%), borderline in 110 (22%) and anxious in 245 (49%). Depression levels were categorized as normal in 218 women (43.6%), borderline in 123 (24.6%) and depressed in 159 (31.8%). The chi-squared test revealed significant associations between the participants’ background and anxiety (χ² = 43.69, df = 4) and depression (χ² = 83.19, df = 4) (both P < .001). This reflects the fact that anxiety was found in 123 (67.6%) of the rural women versus 83 (39.9%) of the urban participants and only 39 (35.5%) of the women with a semiurban background. A similar trend was found for depression, which was observed in 91 (50%) of the women from a rural background, 40 (19.2%) of the urban and 28 (25.5%) of the semiurban women.

Bivariate correlation revealed a significant negative correlation between social support and anxiety (r = −.433, P < .001) and between social support and depression (r = −.453, P < .001). Point biserial correlation showed that the occupations of pregnant women significantly correlated with anxiety (r_pb_ = .17) and depression (r_pb_ = .16) (both P < .001). Employed women reported higher levels of anxiety and depression. A history of harassment, miscarriage, abortion, the number of cesarean deliveries, number of episiotomies and number of unplanned pregnancies were also significantly associated with anxiety and depression (Table 1).

**Table 1:** Significant correlations between socioeconomic and obstetric variables with anxiety and depression in pregnant women (N = 500) surveyed in Lahore, Pakistan, in 2014.

Variable	Anxiety	Depression
Social support	−.43^3^	−.45^3^
Occupation	.17^3^	.16^3^
Harassment	.13^2^	.10^1^
Abortion	.10^1^	.10^1^
Unplanned pregnancy	.23^3^	.28^3^
Cesarean delivery	−.09^4^	−.13^2^
Episiotomy	.15^3^	.10^1^
Vaginal delivery	.10^1^	.07

^1^ P < .05

^2^ P < .01

^3^ P < .001

^4^ Marginally significant

Significant associations were found between modes of delivery, scores on the HADS anxiety and depression subscales, and SPS score (Table 2). Increasing numbers of cesarean deliveries were associated with higher SPS scores (rho = .13, P <.01), and increasing numbers of episiotomies were associated with lower SPS scores (rho = −.10, P < .05).

**Table 2:** Associations between modes of delivery and scores on the Hospital Anxiety and Depression Scale in pregnant women (N = 500) surveyed in Lahore, Pakistan, in 2014.

Mode	Anxiety				Depression			
	Normal	Borderline	Severe	χ^2^	Normal	Borderline	Severe	χ^2^
Episiotomy (n)	18	8	55	15.3^3^	27	20	34	5.48^4^
	22%	9.9%	67.9%		33.3%	24.7%	42%	
C-delivery (n)	51	29	56	7.02^1^	73	32	31	9.20^2^
	37.5%	21.3%	41.2%		53.7%	23.5%	22.8%	

^1^ P < .05

^2^ P < .01

^3^ P < .001

^4^ Marginally significant

Linear regression was used to test whether the number of daughters and sons (as dichotomous variables) and scores on social provisions scale (SPS) successfully predicted scores on the HADS depression subscale (Table 3). For this purpose, two models were created. In the first model (Model 1) the numbers of sons and daughters were entered as predictors. This model yielded statistically significant results (P < .01) that explained 2.2% of the variation in the depression subscale scores. The number of daughter was associated positively with the scores whereas the number of sons was associated negatively with them.

**Table 3:** Multiple linear regression model for variables associated with scores indicating depression on the Hospital Anxiety and Depression Scale in pregnant women (N = 500) surveyed in Lahore, Pakistan, in 2014.

Model	Predictor	B	Standard Error (B)	Beta
Model 1 (R^2^ = .022)	Number of sons	-.982	.366	-.121^3^
	Number of daughters	1.015	.424	-.108^2^
Model 2 (R^2^ = .213)	Number of sons	-.661	.329	-.081^1^
	Number of daughters	.524	.383	.056
	Social support (SPS)	-.146	.013	-.44^2^

^1^ P < .05

^2^ P < .01

^3^ P < .001

When SPS scores were entered into model 2 along with the previously identified predictors (numbers of daughters and sons), the effect size of the model (R^2^) increased to .213, i.e., model 2 explained 21.3% of variation in HADS depression subscale scores. However, SPS scores exerted a strong controlling effect on other predictors, consequently decreasing the **B** values of the number of daughter and sons. The inclusion of SPS scores in model 2 also rendered the association between the number of daughters and HADS depression subscale scores non-significant.

Bivariate correlations revealed that the total number of children (r = .096, P < .05) and number of daughters (r = .128, P < .01) were associated with high anxiety subscale scores. The number of daughters also showed a negative association with scores on the social support scale (r = −.103, P < .05).

The point biserial correlation was significant between the total number of daughters and reported harassment (r_s_ = .11, P < .05).

## Discussion

Our study showed a high prevalence of both antenatal depression (31.8%) and anxiety (49%), which is in consonance with earlier studies conducted in Pakistan. In comparison, studies from developed western countries have generally reported a lower prevalence. For instance, a nationwide survey conducted in the USA from 1996 to 2006 reported a prevalence of antenatal anxiety or depression or other mental health problems of 7.8% [35]. These results underscore the importance of prenatal depression and anxiety as a major public health problem in our country. To address this grave situation, effective screening and intervention methods should be planned.

Studies in western countries generally report a higher incidence of psychiatric disorders in urban populations than rural populations [36]. In contrast, our study found almost twice the prevalence of antenatal depression and anxiety among rural women as among urban and semiurban women. This apparent contradiction may be explained by the unique environmental factors that pregnant women are exposed to in developing South-East Asian countries. In the cultural context of Pakistan, several social factors are worth mentioning. First, there is a very large gap in the standards of living and available facilities between rural and urban communities in developing countries, whereas this gap is not as large in developed countries. In Pakistan, rural areas lack several basic necessities of life including health services, water sanitation, gas, electricity and higher educational facilities [37]. Furthermore, gender discrimination, while common throughout the country, is especially evident in rural communities. Rural women are less independent and play a lesser role in decision making than urban women. Rural settings also have an adverse effect on the mental health of pregnant women [38]. These factors, in our opinion, are important contributors to the greater depression and anxiety among pregnant women in rural settings in our country. Our findings are consistent with the results from two studies of pregnant women in Sindh province, Pakistan, one in a rural community and the other in an urban community. These studies found a significantly higher prevalence of depression among rural pregnant women (60%) [27] than urban pregnant women (39.4%) [39]. Husain et al., in their study of antenatal depression in an urban population in Pakistan, also pointed out that rural settings with greater poverty, inadequate health facilities and less education might be associated with higher rates of antenatal depression and anxiety [40]. Developmental programs in rural communities may help reduce psychological morbidity in rural pregnant women.

An important risk factor for antenatal depression and anxiety in our study was low social support. Pregnant women who perceived low social support had higher rates of both depression and anxiety, and vice versa. In contrast, Hamayun et al. reported no significant association between social support and antenatal depression in Lahore [41]. However, they used a dichotomous (Yes/No) question to assess social support—a multidimensional construct to assess a person’s social network. Because of its multidimensional nature, perceived social support should be subjectively assessed by psychometrically sound instruments. Our finding is reinforced by many earlier studies conducted throughout the world that reported a strong association between social support and antenatal depression and anxiety [16,23,42]. The association between social support and psychological morbidity is hardly surprising since social support has been found to be connected to depression and anxiety not just among pregnant women but in the general population as well [43]. The exact mechanism by which social support affects depression and anxiety remains obscure. However, it is known that low social support can give rise to a sense of isolation and loneliness, which are both strongly associated with poor mental health [44]. In developing countries like Pakistan, low social support is a particular problem, as demonstrated by the fact that it was the strongest predictor of antenatal depression and anxiety in our study (r value of 0.453 for depression and 0.433 for anxiety). The causes of low social support differ in urban and rural communities of Pakistan. Among urban women, the most common causes include verbal and physical abuse by the husband or in-laws, societal restrictions on women, and living in joint family systems [39]. Among rural women, low social support has been found to result from lack of care by the husband, large age differences between the husband and wife, and greater numbers of children [27]. Many of these factors, which seldom occur in developed countries, highlight the need for society-specific interventions in to improve social support and consequently the mental health of pregnant women in Pakistan and elsewhere.

An interesting finding in our study was the correlation between the occupation of pregnant women and antenatal depression and anxiety. In contrast to studies in western populations, which mention employment as a strong protective factor against major depression in pregnancy [45], our study found that pregnant women employed outside the home were actually more depressed and anxious than housewives. A study in Karachi, Pakistan also apparently contradicts our findings by concluding that housewives, in general, are more depressed than working women [46]. Several factors might explain this contradiction. Most of these studies mention education as an important protective factor against antenatal anxiety and depression. Therefore, the lower educational level of housewives compared to working women was associated with higher levels of anxiety and depression. However, our study included respondents from low and lower-middle socioeconomic classes, and 54% of the women in our sample were educated to less than the 10th grade level. So even most of the working women may not have been educated highly enough for their employment status to have a positive effect on their mental health. Secondly, in recent years inflation has increased and socioeconomic conditions have deteriorated in Pakistan, and these changes have led to increased stress and the pressures on working women to meet the economic needs of their household. It is also well documented that greater work stress can precipitate anxiety and depression in employed men and women [47]. This increased stress, combined with the demands of pregnancy, might be responsible for greater depression and anxiety in working women compared to housewives, who are relatively protected from work stress. Finally, another factor might also be operative in the social environment of our country. In many orthodox Pakistani families, most of which belong to lower and lower-middle social classes, working women are highly stigmatized. In this socioeconomic setting, the home is considered the appropriate place for women, and being an obedient wife and a loving mother are considered their appropriate roles. Negative attitudes among relatives towards their work might contribute to depression and anxiety among working pregnant women from the lower and lower-middle social classes who participated in our study; housewives, in contrast, were protected from such discrimination. Nevertheless, more research is required to clarify the relationship between employment outside the home and antenatal depression and anxiety, especially in the cultural environment in Pakistan.

In this study a history of one or more episiotomies and cesarean deliveries was associated with a high incidence of antenatal anxiety and depression. This is in accordance with a study by Kuo S-Y et al. which showed that more than one third of the women undergoing elective cesarean delivery suffered from anxiety, whereas only one fourth of the women had depression several months after the procedure [48]. Although the increasing prevalence of cesarean delivery is a major public health concern in many countries, it is one of the most common obstetric procedures in South Asia. Antenatal anxiety and depression in pregnant women because of a previous cesarean delivery or episiotomy may be due to concerns about her own health, fear regarding the well-being of her developing child and fears regarding another invasive procedure requiring stressful measures such as anesthesia and a relatively large incision. However, there was a significant difference between the incidence of anxiety and depression between women who had undergone at least one caesarean delivery, episiotomy or normal vaginal delivery. In Pakistan, women from low socioeconomic backgrounds generally tend to avoid hospital deliveries because of sociocultural norms (e.g., the belief that vaginal delivery creates an emotional bond with the baby), the large expense, fear of the procedure or of postoperative infection, and insufficient knowledge [49]. Women prefer vaginal deliveries at home in the care of untrained health care professionals called “dai”, and often seek care at hospital emergency departments only for life-threatening complications. In our society, caesarean delivery is usually termed a “bara operation” (a “big operation”) due to fears and associated sociocultural norms that reinforce negative attitudes towards this mode of delivery. Therefore, women with a history of at least one cesarean delivery enjoy significantly higher social support compared to those who have undergone episiotomies and normal vaginal deliveries.

Another interesting finding in our study was the association between history of induced abortion and antenatal depression or anxiety. Women who had undergone induced abortions were found to be significantly more depressed (r_pb_ = .1) and anxious (r_pb_ = .1) than those with no such history. Our finding is consistent with several studies that reported the incidence of mental disorders in women after undergoing elective abortions in general [50,51] and during subsequent pregnancies [26]. However, a systematic review in 2010 of the risk factors for antenatal depression in North America, Europe and Australia found no significant association with spontaneous or elective abortions [18].

This significant association with induced abortions can be explained by the unique religious and cultural environment of Pakistan. This country is home to a number of Islamic sects as well as various cultures and ethnicities. Most of the religious sects in the country vehemently oppose abortion, considering it murder of the fetus. Thus, in the light of Sharia Law, section 338 of the Pakistani Penal Code criminalizes and strictly prohibits induced abortions. According to Pakistani law, whoever causes the abortion of a fetus before his or her organs have been formed, even with the consent of the pregnant woman, is liable to 3 years of imprisonment, or 7 years if the procedure is done after the fetal organs have formed. Abortions are only permitted if pregnancy threatens the woman’s health. This has resulted in a prevailing stigma towards voluntary abortion in Pakistani society. As shown in a study at a tertiary care hospital in Karachi, only 7% (93) of women admitted with post-abortion surgical complications (1534) acknowledged the induction of abortion, while the rest denied it [52]. Despite the religious and legal prohibition, an estimated 890,000 abortions take place in Pakistan annually [53] for several reasons, which include difficulties raising a large family, spouse unemployment, poverty, ill health of the mother, short spacing between pregnancies, and extramarital affairs [54]. In a nationwide study of the fate of unwanted pregnancies in Pakistan, the Population Council (2004) highlighted the situation of female feticide. In this study, one interviewee reported abortion of a female fetus due to financial concerns over the cost of marriage [55]. However, owing to the prevailing stigma, these figures may be underreported in Pakistan.

Due to legal limitations, most abortion procedures in rural and low socioeconomic areas are carried out by untrained health workers and “dais”. Consequently, women often present in hospitals with severe complications [52]. As a result, pregnant women with a history of abortion not only face harassment and stigmatization by society but also fear the complications of the procedure. These factors may contribute to a higher incidence of depression and anxiety. Effective family planning methods and the provision of safer abortion methods may reduce this problem.

Other factors such as harassment, a history of miscarriage and the unplanned vs. planned nature of the pregnancy were also significantly associated with antenatal anxiety and depression, and have been identified repeatedly in earlier studies [16,21].

A novel and important finding in our study is the relationship between the gender of previous children and the level of antenatal depression and anxiety. Having daughters was significantly associated with antenatal depression and anxiety, whereas having sons was a protective factor. Social support mediated this relationship. These results make sense when we take into account the issue of gender discrimination and the preference for male children in South Asia. In Pakistan the family system is predominantly patriarchal. Women are treated as second-class citizens and denied their social rights. Among the consequences of this social structure are honor killings, the bride price and dowry, the disputed status of female testimony, forced marriages and denial of a woman’s right to have a career. Parents view their sons as bread-earners and agents of continuation of the family name, and view their daughters as an economic burden. This is partly due to the tradition of providing a large dowry when a daughter marries, especially in India and Pakistan. The dowry may be in the form of land, money, jewelry or household items. In many wedding ceremonies the dowry is displayed and announced by the bride’s family. A bridal dress in Pakistan, for instance, can cost up to half a million rupees (US$ 8380), and the whole event can cost up to 20 million rupees (US$ 335,000) [56], most of the expenses being paid by the bride’s family. It is probably for these reasons that the rates of female feticide are alarmingly high in the region [57].

Even after birth, sons are given preference over daughters with respect to access to health care and educational opportunities [58]. In this context, the relationship between higher rates of depression and anxiety among pregnant women with more daughters makes perfect sense. Considering societal pressures, pregnant women who have already given birth to one or more daughters are not only concerned about their future offspring’s gender, but are also subject to harassment, taunting and stigmatization by their family and relatives. This highlights how the unique social conditions in Pakistan arising from gender discrimination against females give rise to a significant and previously unacknowledged predictor of antenatal depression and anxiety, i.e., the gender of previous children. We encourage more research to further investigate this novel association. Widespread social and educational reforms designed to reduce gender discrimination may help to decrease the influence of this factor on the psychological well-being of women of child-bearing age.

## Limitations

Causal associations between these risk factors and antenatal anxiety and depression cannot be established due to the cross-sectional design of this study. Convenience sampling and the lack of data for comparison with control groups limit the generalizability of these results to the whole population of Pakistan. The hospitals that participated in this research do not maintain a patient database in their out-patient departments. Therefore, retrospective comparisons with control groups were not possible. The HADS was used in this study to screen for antenatal depression and anxiety in this sample. This instrument has been culturally validated and shown reliable in Pakistan, but is based on criteria that are not complete transposable to the International Classification of Diseases and Related Health Problems (ICD-10) diagnosis of depression or anxiety.

## Conclusion

In the context of the predominantly patriarchal sociocultural setting that characterizes Pakistan, the predictors of antenatal anxiety and depression may well differ from those in developed countries. Rural women and working women in our sample of participants had higher levels of antenatal anxiety and depression, which contrasts with studies from western countries. Our study found that higher numbers of daughters were associated with higher levels of depression and anxiety, whereas higher numbers of sons had a protective influence. We therefore suggest that interventions designed and implemented to reduce antenatal anxiety and depression should take into account these unique factors operating in developing countries and patriarchal societies.

## Supporting Information

S1 Data Dataset for " Psychosocial factors of antenatal anxiety and depression in Pakistan: Is social support a mediator?" in .csv format. (CSV)
